# "It’s About the Idea Hitting the Bull’s Eye": How Aid Effectiveness Can Catalyse the Scale-up of Health Innovations

**DOI:** 10.15171/ijhpm.2018.08

**Published:** 2018-02-14

**Authors:** Deepthi Wickremasinghe, Meenakshi Gautham, Nasir Umar, Della Berhanu, Joanna Schellenberg, Neil Spicer

**Affiliations:** IDEAS Project, London School of Hygiene & Tropical Medicine, London, UK.

**Keywords:** India, Nigeria, Ethiopia, Scale-up, Aid Effectiveness

## Abstract

**Background:** Since the global economic crisis, a harsher economic climate and global commitments to address the problems of global health and poverty have led to increased donor interest to fund effective health innovations that offer value for money. Simultaneously, further aid effectiveness is being sought through encouraging governments in low- and middle-income countries (LMICs) to strengthen their capacity to be self-supporting, rather than donor reliant. In practice, this often means donors fund pilot innovations for three to five years to demonstrate effectiveness and then advocate to the national government to adopt them for scale-up within country-wide health systems. We aim to connect the literature on scaling-up health innovations in LMICs with six key principles of aid effectiveness: country ownership; alignment; harmonisation; transparency and accountability; predictability; and civil society engagement and participation, based on our analysis of interviewees’ accounts of scale-up in such settings.

**Methods:** We analysed 150 semi-structured qualitative interviews, to explore the factors catalysing and inhibiting the scale-up of maternal and newborn health (MNH) innovations in Ethiopia, northeast Nigeria and the State of Uttar Pradesh, India and identified links with the aid effectiveness principles. Our interviewees were purposively selected for their knowledge of scale-up in these settings, and represented a range of constituencies. We conducted a systematic analysis of the expanded field notes, using a framework approach to code a priori themes and identify emerging themes in NVivo 10.

**Results:** Our analysis revealed that actions by donors, implementers and recipient governments to promote the scale-up of innovations strongly reflected many of the aid effectiveness principles embraced by well-known international agreements - including the Paris Declaration of Aid Effectiveness. Our findings show variations in the extent to which these six principles have been adopted in what are three diverse geographical settings, raising important implications for scaling health innovations in low- and middle-income countries.

**Conclusion:** Our findings suggest that if donors, implementers and recipient governments were better able to put these principles into practice, the prospects for scaling externally funded health innovations as part of country health policies and programmes would be enhanced.

## Background

Donor interest in funding health programmes that demonstrate effectiveness and value for money is intensifying, prompted by the global economic crisis and global commitments to address health and poverty problems embodied in the Sustainable Development Goals. One of the common approaches adopted by donors is to fund implementers to undertake innovative pilot health projects and programmes over finite periods to provide evidence of effectiveness, and then advocate recipient governments for their adoption and scale-up within country health programmes and systems. However, as this paper explores, this is challenging to achieve in practice, something others have also commented on.^1-5^

### Scale-up

There is an extensive literature on factors facilitating and undermining the scale-up of innovations in health and other sectors. In this paper, in which we look at innovations piloted using external donor funding, with government support sought for taking them to scale, we define ‘innovation’ as *a community-based approach to enhancing maternal and newborn health [MNH], which may be new or had been introduced elsewhere, but is being implemented in a new context*. These encompass an innovation’s attributes, including simplicity; comparative advantage over alternatives and whether benefits are observable; the ‘receiving environment,’ including the attitudes and needs of potential adopters; and the influence of community opinion leaders and policy champions.^6-15^ Health systems, political, economic and social contexts also influence whether innovations are scaled up including the capacity, training and attitudes of health workers and the strength of commodity logistics and supervision systems onto which new innovations might be layered.^6,9,10,13,16,17^ Decision-makers’ ideas and ideologies often shape which health issues are prioritised and which policies and programmes are financed within the constraints of a country’s economic resources. Innovation adoption by local communities is influenced by health needs, beliefs, sociocultural values and norms, and access, which may be constrained by economic and geographical barriers.^18-21^

We define scale-up as: *government adoption and implementation of health innovations, increasing geographical reach to benefit a greater number of people beyond externally-funded implementers’ programme districts.* We conducted a qualitative study to explore the factors influencing scale-up of MNH innovations in Ethiopia, northeast Nigeria and Uttar Pradesh, India. Specifically, we identified the main actions that implementers can adopt to catalyse innovation scale-up and the influence of geographical contexts on scale-up*.*^4,11^ A strong emerging theme in our analysis, which we report in this current paper, was that donors, implementers and recipient governments’ actions for promoting innovation scale-up also reflected many of the principles embraced by well-known international aid effectiveness agreements. Indeed, these three countries are signatories to the 2005 Paris Declaration of Aid Effectiveness, the 2008 Accra Agenda for Action and the 2011 Busan Partnership for Effective Development Cooperation.^22-25^ Both Ethiopia and Nigeria signed the International Health Partnership Plus (IHP+) Global Compact in 2007,^26^ and have since signed country compacts committing to operationalise effective development cooperation in their health sectors.

### Aid Effectiveness and Health Programmes

For many years, low- and middle-income countries (LMICs) have depended on funds from bilateral donors, global initiatives and philanthropic foundations for implementing health programmes. Yet this method of funding programmes has well-documented problems – often known as problems of aid effectiveness – including poor alignment between what donors fund and recipient countries’ priorities; short-term and unpredictable donor funding; and donors introducing parallel systems and processes for implementing programmes, supplying commodities, and monitoring and reporting programme effects. These factors can burden recipient governments and undermine their ownership of programmes.^27-32^

A desire to improve aid effectiveness, led representatives from LMICs, major donors, leading civil society organisations and United Nations (UN) agencies to agree principles that donors and recipient country governments should adopt. While the terminology used in these agreements varies, they were brought together under the umbrella of the Global Partnership for Effective Development Cooperation (GPEDC) in 2011.^33^ Table 1 provides a summary of six of the major principles embraced by these declarations. An emerging theme from the analysis of our interview data was that these factors were strongly associated with scale-up. It should be noted though, that there are several other aid effectiveness principles including: managing for results; South-to-South cooperation; private sector involvement; and gender equality and women’s empowerment.

**Table 1:** Key Aid Effectiveness Principles^a^

**Aid Effectiveness Principle**	**Definition**
Country ownership	Recipient government involvement, buy-in and leadership of externally funded health programmes and donor programmes working through and strengthening existing country health systems
Alignment	Donors and implementers working in alignment with recipient country priorities, policy frameworks and health systems
Harmonisation	Donors and implementers coordinating programmes
Transparency and accountability	Donor and implementer transparency and harmonised monitoring and evaluation indicators
Aid predictability	Assurance of longer term and more predictable donor funding
Civil society engagement and participation	Government responsiveness to civil society demands

^a^ These principles are included in the Paris Declaration on Aid Effectiveness (2005); the Accra Agenda for Action (2008); the Busan Partnership for Effective Cooperation (2011); IHP+ (from 2007) and the Global Partnership for Effective Development Cooperation (2011).

While there has been progress towards adopting some of these principles, change has not been universal. *‘Some unfavourable practices’* remain,^30^ including ‘vertical’ project funding focussed on specific health issues, rather than supporting broader health system strengthening, and donors continuing to set their own agendas which recipient governments are expected to accept. The Organisation for Economic Co-operation and Development (OECD) also acknowledges that aid predictability and donor harmonisation are often lacking; and governments have not met targets for domestic health expenditure, systems’ strengthening and reforms, or drawn civil society into policy discussions.

This paper aims to connect two fields of study: the scale-up of MNH innovations, that in their pilot phase had been funded by external donors, but where governments were involved in taking them to scale, and principles of aid effectiveness in LMICs. While several studies highlight the importance of innovations aligning with country priorities and that country ownership greatly increases the prospects of governments adopting innovations,^4,6,7,12-15,28^ existing literature has not systematically analysed how donors, implementers and recipient governments’ adherence to aid effectiveness principles affects the scale-up of externally-funded innovations. These innovations sought either to develop existing, or introduce new approaches and many aimed to improve government MNH care services in rural areas (Box 1). Based on our analysis of our interview data, we argue that limited adoption of aid effectiveness principles by donors, implementers and recipient governments weakens the environment for scaling-up externally-funded health innovations.

**Box 1. Examples of Innovations for Improved MNH Services**

- Developing and expanding the roles of frontline health workers (including community volunteers) and introducing incentives and tools to strengthen frontline workers’ performance, including communication materials, mobile phone technologies and quality assurance measures
- Enhancing healthcare referral systems to expand facility deliveries, eg, initiating emergency transport schemes, a MNH call centre, and extending the role of community health workers to include making referrals
- Supporting the community to increase its demand for services, by promoting behaviour change and local decisionmaking

Abbreviation: MNH, maternal and newborn health.

## Methods

We conducted qualitative, semi-structured stakeholder interviews in Ethiopia, northeast Nigeria and Uttar Pradesh, in these three diverse geographical settings, with a common feature of having some of the highest burdens of maternal and neonatal mortality in the world. We worked with researchers trained in qualitative methods, from the three countries and the United Kingdom, to develop a topic guide that we piloted at a workshop in Addis Ababa. After minor adaptations for each country, trained researchers used this guide when conducting 50 interviews for each setting in 2012 and 2013.

The interviewees were purposively selected for their detailed understanding of what is involved in scaling-up MNH innovations funded by donors, and represented government departments, implementers and development partners working on MNH programmes in the settings. Of the donor-funded implementers, most worked for international nongovernmental organisations (NGOs) or large local NGOs. Others were US-based universities and for-profit consultancy agencies. To maintain our respondents’ anonymity, we have not referred to specific organisations in this paper. Table 2 categorises interviewees by broad type, across the three geographical settings. The breakdown of interviewee types was similar in each setting, although in India, where the private sector has a significant role in proving MNH healthcare, we interviewed representatives from that sector.

**Table 2:** Breakdown of Interviewees by Type

**Category of Interviewees**	**Number Interviewed**
Implementers	72
Government officials^a^	25
Donor foundations	8
Bilateral donors	8
Multilateral donors	12
Private sector representatives	3
Academics (university lecturers, researchers and members of professional associations)	22

^a^ Including national government officials in Ethiopia and Nigeria, among them Ministry of Health officials, and state ministry of health officials in Nigeria and Uttar Pradesh.

The MNH projects our interviewees referred to had typically received external funding for three to five years, and varied in scale from a few districts (India), local government areas (Nigeria), or *woredas* (Ethiopia), to many districts across multiple states or regions, and some were part of larger multi-country grants. Some implementers spoke of innovations they had developed which government was now scaling-up, or had incorporated aspects of into government practices; others were preparing innovations for scale-up. Yet mostly, implementers shared the many challenges they had faced when trying, but not succeeding in scaling-up innovations.

All respondents gave informed consent before their interviews. Interviews were conducted in private spaces to ensure confidentiality and, where agreed, were recorded. Soon after each interview, interviewers wrote expanded field notes,^35^ setting out details of the interview under topics reflecting our research questions and emerging themes, and incorporating respondents’ direct quotes. Aid effectiveness issues emerged as a strong theme in our early interviews, which the research team further explored in later interviewees.

By using investigator triangulation to compare and agree researchers’ interpretations, each set of expanded field notes became the work of multiple researchers, thus helping reinforce the validity of the results reported. In addition, researchers from the United Kingdom and the three study countries attended an analysis workshop to reach consensus on interpretations and cross-country comparisons. Systematic analysis of the expanded field notes was conducted using a framework approach to code *a priori* themes and identify emerging themes in NVivo10 as we sought to examine the actions and factors that catalysed scale-up and the contextual factors enabling and undermining it. Aid effectiveness issues emerged in our data analysis which we related to key principles of aid effectiveness. The emerging themes were categorised by two researchers, separately, using an inductive analytic framework based on the aid effectiveness principles. When they were compared, the two analyses mostly concurred, but where there were discrepancies we returned to the data to check the most plausible explanation and sought inputs from co-authors. We also conducted checks by presenting provisional results to interviewees and country stakeholders in Lucknow, Addis Ababa and Abuja, who were invited to comment and confirm the accuracy of our messages.

Our analysis and the quotes we have used reflect a balance of views from the different stakeholder groups, drawing out common views across the full range of stakeholders rather than focusing on a select few. Indeed, our analysis suggested there was considerable agreement on issues between different stakeholder groups.

We received ethics approval from the corresponding author’s institute; the Ethiopian Federal Ministry of Science and Technology; the Regional Health Bureaus of Amhara, Oromia, SNNP and Tigray regions; the Indian Council of Medical Research and SPECT-ERB in India; the Nigerian National Health Research Ethics Committee and Gombe State Ministry of Health.

## Results

We examined ways that embracing each of six key aid effectiveness principles fosters scale-up of health innovations, and compared and contrasted the extent to which this had been achieved in the three settings, including the challenges faced. Table 3 presents some of the key features of the aid effectiveness principles enabling and undermining the scale-up of innovations in each of the three geographies, based on our analysis of all 150 respondents’ accounts.

**Table 3:** Key Features of Aid Effectiveness Principles Enabling (+) and Undermining (-) Scale-up of Innovations

**Aid effectiveness Principle**	**Ethiopia**	**Northeast Nigeria**	**Uttar Pradesh, India**
Country ownership	(**+**) National government coordination of donor-funded programmes fostered government ownership, increasing the possibility of innovations being scaled-up	(**-**) Limited state funding, meant rural primary healthcare was largely donor funded and driven, inhibiting state government ownership and scale-up of innovations	(**+**) State government champions fostered introduction of externally funded innovations, increasing the likelihood of them being scaled
	(**-**) Attrition among government officials made ownership of an innovation transitory		
Alignment	(**+**) Externally funded programmes expected to align with national health strategies and increasingly, implementers supported government work packages, enhancing prospects of innovations being taken to scale	(**-**) Externally funded programmes expected to align with government strategies, but limited government coordination of donor activity meant potentially scalable innovations were missed	(**+**) Economic development in India reduced reliance on external aid; externally funded innovations have had to align with national and state-level government strategies to be considered for scale-up
Harmonisation	(**+**) National government-led *Technical Working Group* on MNH strengthened coordination, reducing duplication of donor-funded innovations and fostering better information sharing	(**+**) Federal government-led *Maternal and Newborn Health Core Technical Committee* encouraged collaboration among some donors to avoid duplication of effort	(**+**) The *Health Partners’ Forum* enabled partner programmes to be mapped to avoid duplication and identify scalable innovations
	(**-**) NGO implementers’ involvement in the *Technical Working Group* limited to responding to technical queries	(**-**) Weak capacity of the government’s *Maternal and Newborn Health Core Technical Committee* to coordinate donor-funded innovations and programmes	(**-**) *Health Partners’ Forum* had limited engagement from donors and leadership from government
	(**-**) Multiple donors and implementers working on parallel health innovations and programmes meant competing interests, priorities and donor-led ways of working, leading to parallel procedures and increased health worker workloads, thus reducing their time for implementation		
	(**-**) Collaboration among implementers was challenging because of their need to claim attribution for innovation outputs as evidence to report to their funders		
Transparency and accountability	(**+**) *Technical Working Group* promoted better transparency and information sharing, which improved understanding of scalability of innovations	(**-**) Transparency hampered by limited government capacity for donor and implementer coordination at federal and state levels	(**+**) *Health Partners’ Forum* was seen as helping to encourage transparency and developing as a space to share information about innovations
			(**-**) *Health Partners’ Forum* was still largely nascent
	(**-**) Parallel donor and implementer monitoring and evaluation and information systems limited opportunities to compare results about innovations and increase understanding		
Aid predictability	(**+**) The pooled *Millennium Development Goals Performance Fund* offered some flexibility for Ministry of Health to fund new innovations at scale	(**-**) Security situation meant donors were becoming reluctant to fund pilot innovations for potential scale-up	(**+**) Relatively high levels of government funding mitigated the negative impact of fluctuations in external funding
	(**-**) Some donors continue to emphasise project-based funding, which is vulnerable to shifting global health priorities		
	(**-**) Short time frames for donor-funded innovations limited the time available to convince government of their value for scale-up		
Civil society engagement and participation	(**+**) By working collectively, civil society organisations were beginning to influence government decisions and priorities for health	(**+**) Civil society was starting to influence government decisions and priorities for health	(**+**) Through the *Health Partners Forum*, government was becoming more responsive to civil society organisations advocating on health needs
		(**-**) Limited awareness of rights undermined civil society organisations’ ability to hold government to account	

Key: (+) = enabler, (-) = barrier.

### Country Ownership: “You Need Government Buy-in at Top, Middle and Bottom”

Country ownership means that government leads a recipient country’s development policies and strategies, to which donors align their funding. It also refers to strengthening government systems through donors’ and implementers’ technical support, and using recipient country systems, rather than introducing parallel ones. Our respondents explained that country ownership is fundamental to scaling-up innovations; government engagement is required at all stages of an innovation’s development, including design, implementation and evaluation. Without it, government would have little interest or stake in an innovation’s success, making its adoption unlikely; *‘Ultimately the owner of scale-up is [national] government…buy-in and ownership within it is important’* (foundation donor, India)*.* Alongside engaging government formally, engendering strong ownership from influential government officials was vital to scale-up. Hence, respondents urged implementers to allow considerable time for dialogue and trust building: *‘…relationship management...with external development partners and the [Ethiopian Ministry of Health] – that’s really important...’* (programme officer, Ethiopia). Government champions helped foster momentum and turned engagement into full government ownership: *‘Having a champion at national or state level helps push things further...’* (implementer, India). In Ethiopia, our interviewees pointed to examples where national government commitment of this nature had contributed to scaling-up externally-funded innovations for community care provision for sick newborns and active management of the third stage of labour.

Our respondents asserted that innovations should be embedded within health systems to have realistic prospects of being scaled. Yet, externally-funded implementers continued to introduce their own procedures since using weak government health systems was seen as delaying implementation, making it difficult for them to demonstrate the impact of an innovation to their funders. Our respondents acknowledged that this approach undermines country ownership, leaving innovations unsustainable and lacking the support required to make scale-up possible: *‘...we create parallel systems...but after the project ends it’s the end of everything...’* (implementer, India).

Our data revealed variations in the extent to which country ownership was potentially achievable across the three countries. Ethiopia’s centrally organised control of donor-funded health programmes at federal level meant implementers required substantial government involvement and support – and with sufficient government interest, rapid scale-up of an innovation was conceivable. In Uttar Pradesh, if an innovation gained the support of influential state-level champions, scale-up was possible through mobilisation of state-level resources. For example, the support of a government official helped foster state government interest in a mobile phone tool to support frontline health workers.

Moreover, donor priorities had less of an influence over policy implementation, particularly where they did not align with state government priorities. In northeast Nigeria, however, respondents suggested that government ownership of externally-funded innovations was more restrained. Although decisions on rural primary healthcare were decided at state and district level, low government prioritisation of rural primary healthcare has led to reliance on relatively high levels of donor-driven support for innovations: *‘Government tends to decrease rather than consolidate funding…to priority areas where donors have shown interest’* (academic, Nigeria). Such limited government ownership often left a vacuum at the end of projects piloting innovations: *‘Ownership and sustainability are not usually achieved - most of the time [implementers] finish and go...’* (academic, Nigeria).

### Alignment: “It’s About the Idea Hitting the Bull’s Eye”

Alignment means the focus of aid fits with recipient countries’ health priorities, policy frameworks and targets – an important step towards fostering country ownership. Our interviewees asserted that alignment was critical to scale-up; donors and implementers must ensure their innovations align closely with key national level health policies, namely the National Health Mission and corresponding state Program Implementation Plans (PIPs) in India, Ethiopia’s Health Extension Program and Health Sector Development Program IV and the Nigerian National Strategic Health Development Plan. Respondents said that if innovations helped address policy issues identified by government, they had a good chance of government adopting and scaling them. For example: a health and nutrition project that was scaled-up in Uttar Pradesh through the Village Health and Nutrition Days; and a voucher scheme to increase demand for RMNCH services in slum areas that was later included in the Uttar Pradesh PIP. *‘It’s much easier to introduce an innovation within government systems than from outside if you want it to be taken up…’* (implementer, India).

Respondents pointed to the perennial problem of implementers responding to global or donor priorities to secure funding for innovations which do not always correspond to country priorities: ‘*Donor coordination is weak - there’s a disconnect between their programmes and [country] needs...’* (government official, Nigeria). Periodic changes in government priorities and policies (often arising from incoming leaders or administrations) meant that implementers struggled to respond if they had committed to fixed deliverables: *‘…there’s a fickleness in the entire system’* (implementer, India).

In Ethiopia, interviewees highlighted the importance of alignment: implementers had to be seen as *‘enabling,’* rather than *‘threatening or questioning [government]’* (project officer); hence they needed to respond to government invitations to develop projects, rather than design innovations and then advocate for government acceptance. Referring to some refresher training for Ethiopia’s Health Extension Workers, respondents noted the importance of the government identifying the need for an innovation and then implementers stepping in to meet it. In India, where economic development and corresponding cutbacks in external aid had reduced donor influence, it was also vital for externally-funded implementers to align their work closely with state priorities to be valued by government, while innovations that created parallel systems would *‘bite the dust’* (government official). In northeast Nigeria by contrast, state governments were described as willing for donors to support MNH programmes with innovations; whether these aligned with government programmes was less of a concern. Yet, as our respondents explained, the fact that health is inadequately funded decreased the prospects of state government adopting and financing an innovation introduced by an externally-funded implementer.

### Harmonisation: ‘Donor Coordination Is Key to any Scale-up’

Harmonising coordination among donors and their implementers includes coordinated implementation of health innovations, and using common systems and procedures. Our interviewees asserted that harmonisation was important for scaling-up innovations since coordinated communication with government enabled government to make informed decisions about scale-up, and shared information on what worked and why helped develop stronger innovations: *‘Don’t re-invent the wheel. Documentation of what worked and lessons learnt is very important’* (implementer, Nigeria). Additionally, interviewees stressed that poor harmonisation put pressure on the health system. One example is where health workers’ workloads increased when donor innovations and programmes introduced parallel procedures – thereby limiting their capacity to implement programmes at scale: *‘Every new programme...you have a new set of forms...that adds a lot of workload...’* (implementer, India).

Despite wide acknowledgment of the importance of harmonisation, our respondents reported perennial problems making it difficult to achieve in practice. These included the large number of donors and implementers working on parallel innovations and programmes, the continued focus on ‘vertical’ project funding, and donors’ competing interests, priorities and ways of working. Implementers were competing for finite donor financial resources, were under pressure to deliver results within ambitious timeframes and attribute outcomes to specific programmatic efforts that were easier to measure if working alone:* ‘Who takes the credit for the outputs and outcomes when donors leverage resources to implement programmes together?’* (implementer, Nigeria).

Key to strengthening harmonisation between innovations was the effective functioning of government-led donor coordination mechanisms and the willingness of stakeholders to embrace them. Specifically, a *Technical Working Group* responsible for MNH in Ethiopia, the *Health Partners’ Forum* in Uttar Pradesh and Nigeria’s *Maternal and Newborn Child Health Core Technical Committee.* Our respondents highlighted differences across the three settings. In Ethiopia, interviewees acknowledged donor harmonisation was relatively strong; the Technical Working Group was becoming institutionalised, and had encouraged improved donor engagement and communication, although, interviewees also suggested that implementers’ involvement was often limited to providing technical inputs when requested. In Nigeria, partial donor and implementer harmonisation posed challenges to scaling-up innovations: *‘Everybody is doing things in isolation; there’s no proper coordination…’* (implementer)*.* Yet there were some reports that the donor forum was developing: *‘...integration among donors has improved...but there’s still a lot to be done...’* (implementer, Nigeria). In Uttar Pradesh, while the Health Partners’ Forum was nascent, mapping partner innovations and programmes had strengthened harmonisation, although a few respondents criticised donors for their partial engagement and state government for its leadership: *‘...the kind of leadership required to make things move smoothly is not there’* (implementer, India).

### Transparency and Accountability: “…Trust Among Decision-Makers and Care Providers”

Transparency includes donors being open about their programmes and their impacts, and providing recipient governments with data that aligns with national health information, thereby improving their accountability to those governments. Such transparency can foster scale-up since better information flows from donors and implementers to government, and coordinated information flows among multiple donors and implementers strengthens government’s ability to make informed decisions about potentially scalable innovations. Further, better information sharing about innovations with government tends to foster trust, which interviewees suggested put development partners in a stronger position to advocate for their innovations to be scaled. This happened for a community care of sick newborns innovation in Ethiopia and a post-abortion care innovation in Nigeria. In the Nigerian example, an international NGO was transparent about evidence it found showing that a shortage of doctors able to administer post-abortion care in Nigeria was creating a backlog of women needing care, and then advocated for an innovation to train nurses in post-abortion care to help meet the need. Through being transparent about the evidence collected at each step, trust was built with policy-makers leading them to accept the link between this training and shorter waiting lists, which subsequently led to the inclusion of post-abortion care in the curriculum for nursing students so that the task shifting became institutionalised.

In practice, implementers routinely established parallel monitoring and evaluation and information systems for innovations, rather than using - and potentially strengthening - government systems. Yet, respondents reported some progress towards better transparency. Ethiopia’s Technical Working Group was described as enabling transparency - which increased the chances of innovations being scaled-up: *‘[It’s] the most important enabling factor’* (implementer, Ethiopia). Similarly, respondents reported that the Uttar Pradesh Health Partners Forum was starting to* ‘encourage transparency’* (implementer, India) and evolving into a *‘sharing platform’* (multilateral donor, India) about innovations, from which *‘coordination between various actors…is likely to come’* (implementer, India)*.* In Nigeria, attempts to improve transparency – including strengthening federal and state-level mechanisms for coordinating innovations – were described during the interviews as in their infancy. Nevertheless, the intention to improve transparency existed: *‘It’s no longer business as usual. People are asking questions, people want to be informed’* (multilateral agency, Nigeria).

### Predictability: “Programmes Run Only as Far as Funding Is Available”

Predictability includes donors being clear about how long governments can expect to receive funding and about anticipated future funding for an innovation, and also where possible, governments and implementers diversifying funding sources to create financial stability, so that innovations can continue and be scaled up. This may be more possible in middle-income countries like India and Nigeria, than in Ethiopia, which receives substantial external funding, but without some financial stability, it is difficult for recipient governments to plan long-term health spending commitments - including financing innovations introduced by externally-funded implementers. Many interviewees highlighted the advantages of longer term and more predictable donor grants that, if forthcoming, would allow implementers to include a proper project planning phase, time and resources for effective advocacy and other scale-up activities, and for the innovation to develop and mature. It could also allow for committed time to support government to implement innovations at scale.

In practice, short-term grants are common for MNH innovations, with timely commitments to future funding often unclear or unpredictable. Hence, implementers were in a permanent cycle of applying for funding, delivering projects under pressure and requesting no-cost extensions, or seeking funding from alternative donors to maintain services they had developed. Such grants were challenging for scale-up, providing limited time to convince government of an innovation’s value: ‘*By the time the programme starts showing results it is finishing’* (implementer, India). Additionally, erratic funding commonly led to interruptions in innovation services, which interviewees described as impacting negatively on community experiences of the health system, thereby jeopardising future prospects of scaling-up innovations: ‘…*when you create demand and there’s no supply you have people who are disillusioned - people who feel betrayed are not willing to access the system anymore’* (bilateral donor, Nigeria)*.*

Problems of predictability varied between the settings. In Uttar Pradesh, relatively high levels of government funding for rural primary healthcare meant that fluctuations in donor funding had minimal impact – although implementers still faced the problems of short-term funding for innovations. In northeast Nigeria, donor projects had previously been supporting rural primary healthcare substantially, but the security situation in the region had led some donors to discontinue funding for innovations, because of the difficulty of achieving results in such circumstances. Speaking about the insurgency, a Nigerian government official said: *‘Donors are unwilling to go to areas considered as hot spots with a lot of uncertainties.’* In Ethiopia, domestic resources were more limited, with substantial external aid required to maintain health innovations, which were vulnerable to shifting global priorities: *‘…most innovations…succeed in their pilot phase, because of intensive resources…but not many do so when it comes to scaling implementation, after funders have pulled out’* (implementer, Ethiopia). To partially overcome funding uncertainties, some donors were contributing to Ethiopia’s pooled ‘Millennium Development Goals Performance Fund.’ This gave the government some flexibility to fund promising new innovations: *‘… [it] enables the ministry to fill gaps in their own health development programme,’* noted a bilateral donor, whose agency had decided that this was the best way to assist government efforts in the health sector.

### Civil Society Engagement and Participation: “People Are Beginning to Make Demands”

The recognition of civil society as key development actors is promoted in international aid effectiveness agreements.^22,23^ Our respondents explained that civil society engagement in decision-making bodies such as donor coordination mechanisms reinforced efforts to scale innovations by giving NGOs a platform to advocate to government. If government is receptive to civil society this can mean it is more likely to respond favourably; indeed, a vibrant civil society is well placed to raise the profile of health issues such as MNH and encourage government to increase attention and financing.

Interviewees in northeast Nigeria noted that NGO implementers were becoming participants in state-level decision-making with government, including decisions about allocating resources to MNH programmes and innovations: *‘There’s a gradual shift by government in recognising the importance of citizens’ participation in decision-making within a democratic setting’* (implementer, Nigeria). In Uttar Pradesh, interviewees suggested that the political system was also becoming more responsive to civil society; the Health Partners’ Forum helped NGO implementers to start influencing health policy:* ‘...civil society is now viewed as a force of change’* (academic, India). Similarly, civil society in Nigeria had a role in influencing some high-profile health decisions: *‘The democratic space is now open for [civil society] to speak on issues, unlike in the past’* (implementer, Nigeria)*.* Nevertheless, interviewees acknowledged that in that setting civil society’s ability to hold government to account was taking time: *‘many local* [civil society organisations] *are not aware of their power to hold government accountable’* (implementer, Nigeria). Civil society in Ethiopia is not as strong as in Nigeria and India, yet NGO implementers were described as starting to influence government if they worked together; *‘...collectively they can make a policy change...*’ (multilateral donor, Ethiopia), or invoked more powerful actors such as UN agencies to help raise the profile of their work.

## Discussion

We found that the principles of aid effectiveness had a strong bearing on the scaling up of innovations in three very different economic and policy settings, raising important implications for scaling health innovations in LMICs. Where our interviewees noted positive examples of aid effectiveness these were mostly emerging trends that if strengthened would enhance the environment for scale-up. In contrast, poor adherence to aid effectiveness, such as lack of harmonisation, and unpredictability of aid reduced the likelihood of scale-up of donor-funded health programmes, raising questions about the value of short-term, donor-funded health innovations that are unlikely to have lasting substantial impact beyond implementation districts and timeframes. Moreover, short-term health innovations that *‘bite the dust’* could be damaging if they placed an unnecessary burden on weak country health systems, including the workloads of frontline health workers, and led to communities being *‘disillusioned’* by health services dependent on erratic external funding.

We identified key ways in which each of the three main groups of actors ― implementers, governments and donors ― may enhance the prospects of scaling up MNH innovations, not only through their own actions, but also through working together.

### Implementers

The steps which health programme implementers take to maximise the chances of their innovations being scaled are highlighted in existing scale-up literature.^4,6,7,10,12-15,28^ Implementers’ flexibility to respond to changing policy directions helps maximise alignment, a point also raised by Bhutta and Aleem,^27^ and something that donors might consider when deciding which groups to work with. While implementer actions and approaches, and the effectiveness of the innovation itself, are critical factors for scale-up, this paper also highlights the important role of recipient governments and donor agencies.

### Government

Our data show how recipient governments can establish conditions that enable scale-up including: taking ownership of externally-funded innovations by engaging in their design and development; taking strong leadership of donor coordination mechanisms to improve alignment, harmonisation, transparency and accountability; and being responsive towards civil society. Adoption varied across our study settings, perhaps unsurprising given their markedly different economic and political contexts.

Ethiopia, classified by the World Bank as a low-income country eligible for concessional loans,^36^ is heavily reliant on external funding for its health system. While health policy Decision-making lies with national government, health system administration is being decentralised to woreda-level. The Ethiopian Government’s high prioritisation of rural MNH, together with strong Ministry of Health leadership over donor programmes, despite the country being highly reliant on external health funding, meant implementers needed to seek high levels of government ownership to implement any health innovations. Additionally, the Technical Working Group was reasonably strong in coordinating donor health programmes and encouraging information sharing. These emerging factors increased the prospects of government scaling externally-funded health innovations, although a relatively weak civil society had limited influence over government’s decision-making.

In contrast to Ethiopia, India is classified as a lower-middle income country, with the financial ability to borrow interest-bearing loans from the International Bank of Reconstruction and Development (IBRD).^36^ While the Indian Government makes over-arching national policy decisions, the devolved health system, means that individual states have autonomy over local policy implementation and decide how disbursements from central funds are spent. In Uttar Pradesh, high prioritisation of MNH in rural areas through the National Health Mission provided substantial state resources for funding the scale up of selected health innovations, with some backing from influential state level champions, whereas low dependency on external funding meant donors had limited influence over health policies. The emergent, government-led Health Partners’ Forum was starting to improve donor coordination including better information sharing about innovations, while civil society was described as becoming a *‘force for change’* in influencing state government.

Nigeria too is classified as a lower-middle-income country, yet its low per-capita income means that it is eligible to receive both concessional loans from the International Development Association and interest-bearing loans.^36^ The Nigerian, the health system is partially decentralised, in that in some states, the state ministries of health make policy decisions, yet delays in the disbursement of funds often hamper implementation. In northeast Nigerian states, rural primary healthcare was considered largely donor-driven and funded. Reported problems of financing not following government commitments undermined the prospects of state governments adopting externally-funded health innovations, although like Uttar Pradesh, civil society had started to influence government decisions in some states.

### Donors

Donor agencies have an important role in the scale-up of innovations since they have substantial influence over implementer behaviour. If donors choose to fund health issues that do not align with country-defined priorities, or continue to support vertical projects rather than broader health systems work, there is little to encourage country ownership.^27,30,32^ Moreover, donor decisions affect the predictability of funding for innovations, in terms of whether they focus on shorter-term projects, are willing to fund longer-term programmes, or contribute to pooled health sector funding.^30,37^ Donors’ demands on their implementers have implications for scaling-up innovations, including expectations about achieving results within ambitious timeframes, and how implementers report to them, what indicators are reported and whether country monitoring and reporting systems are embraced.^37^ Donors can contribute to transparency by making apparent what they are funding, the impacts of their programmes and stimulating transparency among their implementers.^30,32,37^ Beyond funding, donors can also: assist national governments to strengthen their capacity for ownership for innovations, avoid duplicating procedures, and harmonise with recipient country processes,^22^ ensure alignment with national priorities and a long lead-in for planning an innovation’s transition to government.^3^

### Limitations

Our paper focusses on the aid effectiveness principles as key factors influencing the scale-up of innovations. Yet, there are multiple other factors – including the scalability of the innovations themselves, the capability of government to adopt and scale the innovation, embedding scale-up in project plans and generating and presenting robust evidence effectively.^4,11^ While it may not be possible to generalise our findings beyond our focus settings, they offer a useful snapshot in time of three contrasting settings. We have also generalised about highly diverse set of donors and implementers, in terms of their focus and approach, as going into this in more detail was beyond the scope of this study. Additional research would be of value to understand these issues in other settings and unpack different approaches to influence scale-up.

The nature of qualitative research means that the questions asked in interviews and the way they were analysed and interpreted may have be prone to some degree of subjectivity. Counterbalancing this, the researchers were external to the projects and thus had no specific interest in presenting them in a positive or negative light, which we hope has meant that our reflections are objective.

## Conclusion

Our findings offer insights into how country ownership, alignment, harmonisation, transparency and accountability, predictability, and civil society engagement and participation can enhance the environment for scaling-up MNH care innovations in diverse settings, despite limitations and variations in the practical implementation of the different aid effectiveness principles outlined. Our study suggests that the links between these six principles of aid effectiveness and the scale-up of healthcare innovations are explicit to each of the three settings and would merit further investigation, across a wider range of countries to draw out specific considerations for recipient governments, donors and implementers.

## Acknowledgements

We would like to thank the country researchers in Ethiopia, India, and Nigeria, who contributed to the data collection for this study. This study was supported by IDEAS - Informed Decisions for Actions to improve MNH (http://ideas.lshtm.ac.uk), which is funded through a grant from the Bill and Melinda Gates Foundation, USA to the London School of Hygiene & Tropical Medicine, London, UK. (Gates Global Health Grant Number: OPP1017031).

## Ethical issues

Ethical approval for this study was granted by the London School of Hygiene & Tropical Medicine, London, UK; the Ethiopian Federal Ministry of Science and Technology, Addis Ababa, Ethiopia; the Regional Health Bureaus of Amhara, Oromia, Southern Nations, Nationalities and Peoples’ and Tigray regions, in Ethiopia; the Indian Council of Medical Research and SPECT-ERB in India; the Nigerian National Health Research Ethics Committee and the Gombe State Ministry of Health, in Nigeria.

## Competing interests

Authors declare that they have no competing interests.

## Authors’ contributions

DW contributed to the analysis, wrote this paper and made critical revision of the manuscript for important intellectual content. NS was the research lead for the study on which this paper is based and contributed to the analysis and writing of this paper, and made critical revisions of the manuscript for important intellectual content. MG, NU, and DB made critical revisions of the manuscript for important intellectual content and shared their local expertise about the three geographical settings. JS made critical revisions of the manuscript for important intellectual content and is Principal Investigator for Phase 1 of IDEAS.

## Key messages

**Implications for policy makers**

- By embracing the principles of aid effectiveness and cooperation, policy-makers, donors and implementers of health innovations will enhance the prospects of government being able to take those innovations to scale and bring about improvements in health services and systems.
- Policy-maker engagement throughout the process of scaling-up health innovations is key to ensuring that previously externally funded innovations fit with national health priorities, policy frameworks and targets.
- Government commitment to engage and work with donors and implementers can help to realise the aid effectiveness principles and improve health systems and services in low and middle-income countries, but this will take time.

**Implications for the public**

The internationally agreed aid effectiveness principles encourage greater cooperation between donors, implementers and governments that receive aid and can help those governments to adopt and expand the reach of health service innovations for mothers and babies, which received pilot funding from external sources. Such cooperation will contribute to providing health services that meet the needs of the population and improve public health. The principles include government ownership of innovations, aligning them with national health priorities, and coordinating donors and implementers. Moreover, if these three groups can develop a trusting relationship, through sharing information and creating transparency and accountability, it enhances coordination. Added to this, is recognition of the important contribution civil society can make through working with government to identify local health priorities and feasible ways to address them.
